# Respiratory virus detections in children presenting to an Australian paediatric referral hospital pre-COVID-19 pandemic, January 2014 to December 2019

**DOI:** 10.1371/journal.pone.0313504

**Published:** 2025-01-22

**Authors:** Rebecca Burrell, Gemma L. Saravanos, Alison Kesson, Kin-chuen Leung, Alex C. Outhred, Nicholas Wood, David Muscatello, Philip N. Britton

**Affiliations:** 1 Sydney Medical School, University of Sydney, Sydney, New South Wales (NSW), Australia; 2 Centre for Paediatric and Perinatal Infection Research, The Children’s Hospital at Westmead, Westmead, NSW, Australia; 3 The University of Sydney Infectious Diseases Institute (Sydney ID), University of Sydney, Sydney, NSW, Australia; 4 Susan Wakil School of Nursing and Midwifery, University of Sydney, Sydney, NSW, Australia; 5 Department of Infectious Diseases & Microbiology, The Children’s Hospital at Westmead, Westmead, NSW, Australia; 6 School of Population Health, University of New South Wales, Sydney, NSW, Australia; The University of Hong Kong, CHINA

## Abstract

Acute respiratory infections cause significant paediatric morbidity, but for pathogens other than influenza, respiratory syncytial virus (RSV), and SARS-CoV-2, systematic monitoring is not commonly performed. This retrospective analysis of six years of routinely collected respiratory pathogen multiplex PCR testing at a major paediatric hospital in New South Wales Australia, describes the epidemiology, year-round seasonality, and co-detection patterns of 15 viral respiratory pathogens. 32,599 respiratory samples from children aged under 16 years were analysed. Most samples were associated with a hospital admission (24,149, 74.1%) and the median age of sampling was 16 months (IQR 5–53). Viruses were detected in 62.9% (20,510) of samples, with single virus detections occurring in 73.5% (15,082) of positive samples. In instances of single virus detection, rhinovirus was most frequent (5125, 40.6%), followed by RSV-B (1394, 9.2%) and RSV-A (1290, 8.6%). Moderate to strong seasonal strength was observed for most viruses with some notable exceptions. Rhinovirus and enterovirus were detected year-round and low seasonal strength was observed for adenovirus and bocavirus. Biennial seasonal patterns were observed for influenza B and parainfluenza virus 2. Co-detections occurred in 5,428 samples, predominantly with two (4284, 79.0%) or three viruses (904, 16.7%). The most common co-detections were rhinovirus-adenovirus (566, 10.4%), rhinovirus-enterovirus (357, 8.3%), and rhinovirus-RSV-B (337, 7.9%). Ongoing pan-pathogen surveillance, integrating both laboratory and clinical data, is necessary to assist in identification of key pathogens and combination of pathogens to support effective preventative public health strategies and reduce the burden of paediatric respiratory infections.

## Introduction

Acute respiratory infections (ARI) are a major cause of illness and hospitalisation globally, particularly among young children. Lower respiratory tract infection (LRTI), a subset of ARI including bronchiolitis and pneumonia, were the second leading cause of mortality in children under 5 years of age in 2019 [1]. In New South Wales, Australia, LRTI is the leading cause of communicable disease hospitalisations across all age groups [2], and over one fifth of paediatric ICU admissions in Australia and New Zealand are attributable to LRTI [3]. The burden of disease is not equally distributed across the population with children aged under five years disproportionally impacted. Incidence of ARI is highest in boys aged under 5 years (6.5 cases/person per year; 95%CI 5.2–7.9) and girls aged 5–9 years (6.2 cases/person per year, 95%CI 4.8–7.6), and then declines with age [4]. Children aged under five years experience ARI at more than three times the rate of the general population [5]. The high rate of infection and high disease burden caused by ARI, particularly among children, highlights the need to better understand the pathogen-specific epidemiology of ARI to improve burden estimates and inform more targeted prevention and other intervention strategies.

Influenza and respiratory syncytial virus (RSV) are leading viral causes of paediatric ARI [6–9]. Other viruses are increasingly recognised to contribute to the burden of paediatric ARI, including SARS-CoV-2, human rhinovirus (HRV), adenovirus (AdV), human parainfluenza virus (hPIV), endemic human coronaviruses (HCoV), and human metapneumovirus (HMPV) [10–12]. The burden of many viral causes of ARI remains understudied and there is a need for a pan-pathogen/viral approach to improve knowledge and surveillance. This gap was highlighted during the altered epidemiology of respiratory viruses and other respiratory pathogens in the context of the COVID-19 pandemic and public health responses [13].

Traditional methods for determining viral causes of ARI include antigen detection assays and serological testing, with viral culture viewed as the ‘gold standard’ for many decades [14, 15]. These methods have been replaced by nucleic acid amplification testing (NAAT), such as polymerase chain reaction (PCR), in many settings due to improved turn-around-times, sensitivity, and specificity [14, 15]. The development of multiplex PCR panels compounds these benefits enabling testing for multiple pathogens simultaneously [14, 15] and substantially improves cost efficiency compared to monoplex PCR [14].

In this retrospective, descriptive time series analytical study, we used results from multiplex PCR testing of respiratory samples from children attending the largest children’s hospital in Australia’s most populous state, New South Wales. We aimed to describe the epidemiology, seasonality, and co-detections of respiratory viruses in children prior to the onset of the COVID-19 pandemic.

## Methods

### Study population

We included all laboratory test results from children presenting to the Children’s Hospital at Westmead (CHW) in Sydney, Australia, aged under 16 years were included in this retrospective study. This includes inpatients, emergency department presentations, and outpatients. In these settings, children presenting with any acute respiratory symptoms are routinely tested for respiratory viruses, or at the treating clinician’s discretion. Upper (nasopharyngeal aspirates, nasopharyngeal swabs, throat swabs, and combined nasopharyngeal/throat swabs) and lower (lung aspirates, bronchoalveolar lavages, endotracheal aspirates, bronchial brushing, and sputum samples) respiratory tract samples were included.

### Data sources

Individual line-listed multiplex respiratory pathogen real-time PCR test data and associated demographic and admission data were extracted from laboratory and electronic medical records (Cerner Pathnet and Powerchart) for the period 22/09/2013 to 31/06/2020 (pathogen-specific test n = 940 286). Due to the large number of results, data were extracted in batches on 01/06/2020 and 05/06/2020. Test result data from 2020 were extracted on 14/03/2022. In September 2013, CHW changed its pathology record system. Clinical data in the old dataset were archived and not accessible to the authors. Further, large shifts in testing practices and health-seeking behaviors in early 2020. These data for pre 2014 and post 2019 are thus not included in the present study. Data was anonymized post extraction and prior to analysis by study staff.

### Exclusion criteria

Samples were excluded if they had incomplete testing or admission data, were from individuals aged over 16 years, or were sampled outside of the study period (prior to 2014 or post 2019). Test results were excluded if they were marked as invalid or inhibited in Pathnet by laboratory staff, were results from non-PCR testing, or were PCR results from bacterial or influenza subtype testing. Duplicate results were also excluded.

### Assay details

During the study clinical respiratory samples were tested using one of two commercially available multiplex PCR assays; Seegene Seeplex Respiratory Virus 15 (RV15; Sep 2013—Feb 2018) and Seegene Allplex 26 Respiratory Panel (AP26; Mar 2018 –Jun 2020). Both assays detect and differentiate between the following viruses: AdV, human bocavirus (HBoV; serotypes 1–4), HCoV OC43, enterovirus (EV), HMPV, influenza virus A, influenza virus B, hPIV 1, 2, 3, and 4, RSV-A, RSV-B, and HRV (A/B/C). The RV15 assay detected HCoV 229E and HCoV NL63 as a single target, whilst the AP26 assay detected them separately. A combined variable was created for HCoV 229E/NL63 to allow comparison alongside other viruses over the full study time period (results from AP26 testing were marked as positive if either HCoV 229E or HCoV NL63 was detected, and negative if neither were detected).

### Analysis

Following extraction, data were cleaned and analysed using Python (v.3.11.6). Main libraries used were pandas (v.2.1.1) and numpy (v.1.26.0). Data was visualised using matplotlib (v.3.8.0), seaborn (v.0.13.0), geopandas (v.0.14.0), and upsetplot (v.0.8.0).

### Descriptive analysis

Descriptive analysis of demographic and clinical characteristics of tested children was performed. This included analysis of age, sex, Index of Relative Socioeconomic Disadvantage (IRSD) [16], discharge location (inpatient, emergency department, outpatient), length of stay (LOS), time to sampling, and location of sampling (upper, lower, other).

Frequency and percentages are reported for categorical variables, and median and inter quartile range (IQR) for continuous variables unless otherwise specified. Analyses are reported for all samples, and for sub-groups of interest which included samples with any virus detected, with no virus detected, and with a single virus detected.

### Seasonal curve generation

Cohort time course analyses were smoothed using a 5-week moving average percentage of samples testing positive samples by epidemiological week. Average annual seasonal curves were generated for each virus and show mean percentage positive and 95% confidence interval using seaborn’s lineplot function. Temporal patterns were assessed by visual inspection.

### Strength of seasonality analysis

Seasonal strength was categorised using a modified version of a previously published method [17]. First, a 13-week moving average was applied to weekly frequencies of detections by virus to reduce the impact of non-seasonal outliers. Second, smoothed weekly counts were normalised across each year of the study by calculating the proportion that each weekly count contributed to the total frequency of positive samples within the year. Third, these results were averaged across the full study period to determine average strength of seasonality for each virus. The range of weekly percentage positive across all years year was used to determine the strength of seasonality. Range cut-offs for low, moderate, and high seasonality were based on those used by Muscatello (2019): low (<2.5 percentage points), moderate (2.5–5.0) and high (>5.0) [17]. To determine biennial seasonal strength, the method was then repeated across only even (2014, 2016, 2018) or odd (2015, 2017, 2019) years. The difference between even and odd years was used to determine biennial seasonality, viruses with a difference in range between even years and odd years of greater than one percentage point were determined to be biennial. Final designation of seasonality was determined as cycle length (annual versus biennial) and the associated seasonal strength. In instances of biennial seasonality, the cycle with the largest range was given. Due to an unseasonably early and large influenza season experienced in 2019 [18], children were tested using a Cepheid GeneXpert influenza rapid test and if positive were not usually tested again using the full multiplex assay. As such there was likely underestimation of influenza cases during 2019 using multiplex results alone.

### Co-detection analysis

Count and percentage of each virus detected, and associated co-detections, were described. Heatmaps were generated for samples positive for only two viruses (dual detections) using seaborn’s heatmap function. The percentage heatmap was generated by dividing each row by the total number of dual positive samples with that virus. An upset plot was generated for all multi-positive samples, limited to the top 50 combinations. Combinations were described by frequency of occurrence and number of viruses detected.

### Ethics

This study was conducted with ethics approval from the New South Wales Population and Health Service Research Ethics Committee (2019/PID14498, 2019/ETH12952).

## Results

### Demographic and clinical characteristics

Following test and sample exclusions, a total of 32,599 respiratory samples from children aged under 16 years remained and were included in analyses (S1 Fig). Most samples were collected from children who resided in New South Wales (31,152, 95.6%), with the majority associated with postcodes in Greater Sydney (29,222, 89.64%; S2 Fig). Median age at sample collection was 16 months (IQR 5–53) and there was a slight male predominance (M:F ratio 1.34:1) (Table 1). Children aged 1–4 years (11,741, 36.0%) and infants <6 months (8,852, 27.2%) were the largest age groups in the sample. Approximately one third (10,168, 31.2%) of all samples collected were from children residing in postcodes in the highest IRSD quintile, whilst 5,914 (18.1%) were from the lowest quintile.

**Table 1:** Summary of full dataset.

		Full Dataset	Any Virus Positive	Virus Negative
	**Count (% Full Dataset)**	32599 (100.0)	20510 (62.9)	12089 (37.1)
**Sex**	**Male (%)**	18606 (57.1)	11828 (57.7)	6778 (56.1)
**Age (months)**	**Median (IQR)**	16 (5–53)	14 (5–38)	26 (4–85)
**Age Group**	**<6 months**	8852 (27.2)	5490 (26.8)	3362 (27.8)
	**6–11 months**	4662 (14.3)	3583 (17.5)	1079 (8.9)
	**1–4 years**	11741 (36.0)	8183 (39.9)	3558 (29.4)
	**5–9 years**	4320 (13.3)	2109 (10.3)	2211 (18.3)
	**10–15 years**	3024 (9.3)	1145 (5.6)	1879 (15.5)
*IRSD	**Lowest Quintile**	5914 (18.1)	3906 (19.0)	2008 16.6)
	**2nd Quintile**	5159 (15.8)	3204 (15.6)	1955 (16.2)
	**3rd Quintile**	3761 (11.5)	2300 (11.2)	1461 (12.1)
	**4th Quintile**	6822 (20.9)	4518 (22.0)	2304 (19.1)
	**Highest Quintile**	10168 (31.2)	6188 (30.2)	3980 (32.9)
	**Overseas (N/A)**	775 (2.4)	394 (1.9)	381 (3.2)
**Discharge Location**	**Inpatient**	24149 (74.1)	14262 (69.5)	9887 (81.8)
	**Emergency Department**	6711 (20.6)	5243 (25.6)	1468 (12.1)
	**Outpatient or Other Location**	1739 (5.3)	1005 (4.9)	734 (6.1)
**^Length of Stay (days)**	**Median (IQR)**	3 (1–13)	2 (1–5)	7 (2–47)
**Time to Sampling (days)**	**Median (IQR)**	1 (0–3)	1 (0–1)	2 (0–19)
^**$**^ **Respiratory Tract Sampling Location**	**Upper Tract**	31055 (95.3)	19929 (97.2)	11126 (92.0)
	**Lower Tract**	1347 (4.1)	509 (2.5)	838 (6.9)
	**Other**	197 (0.6)	72 (0.4)	125 (1.0)

Table Footnotes: Total frequency and percentage of column total (n (%)) shown unless otherwise stated.

*IRSD is a general socio-economic score providing insight into socio-economic disadvantage. Those in the lowest quintile represent individuals from areas which many individuals have low access to resources and reduced abilities to participate in society.

^Length of stay was calculated for inpatients only. $Respiratory tract sampling location was grouped according to where in the respiratory tract the tested sample originated. Upper tract samples included nasopharyngeal aspirates, nasopharyngeal swabs, throat swabs, and combined nasopharyngeal/throat swabs. Lower tract samples included lung aspirates, bronchoalveolar lavages, endotracheal aspirates, bronchial brushing, and sputum samples. ‘Other’ samples included respiratory samples without definite categorisation.

Abbreviations: IQR: inter quartile range, IRSD: index of relative socio-economic disadvantage, N/A: Not Applicable

Most samples were associated with an inpatient hospital admission (24,151, 74.1%), followed by an emergency department presentation (6711, 20.6%), then an outpatient clinic (1739, 5.3%). Median time from presentation to sampling was 1 day (IQR 0–3). Median length of stay for inpatients was 3 days (IQR 1–13). Most samples were collected from the upper respiratory tract (31,064, 95.3%).

A total of 20,510 (62.9%) samples tested positive for at least one virus, and the remaining 12,089 (37.1%) samples had no virus detected (Table 1). Compared to virus positive samples, negative samples had a higher median age at sampling (26 months, IQR 4–85 vs 16 months, IQR 5–53), were more frequently taken from the lower respiratory tract (6.9% vs 2.5%), were more frequently associated with inpatient admission (81.8% vs 74.1%), and had a longer median length of stay (7 days, IQR 2–47 vs 3 days, IQR 1–13).

### Single positive detections

Of all positive samples, most were positive for a single virus (15082, 73.5%) (Table 1). Of these single positive detections, HRV was the most frequently detected pathogen (6125, 40.6%), followed by RSV-B (1394, 9.2%), and RSV-A (1290, 8.6%) (Table 2). Parainfluenza viruses 1 (303, 2.0%), 4 (214, 1.4%), and 2 (119, 0.8%) were least frequently detected. Viruses were detected most frequently in children aged 1–4 years. Notable exceptions to this include RSV-B and RSV-A where approximately half of detections for each virus were in children aged less than six months of age (698/1394, 50.1%, and 617/1290, 47.8% respectively). Single positive samples where influenza B was detected were most frequently seen in children aged 5–9 years (133, 37.1%) and had a much higher median age of detection (70 months, IQR 39–107). This was also true of single positive influenza A detections (47 months, IQR 19–84), although less pronounced (Table 2). Influenza B (146, 40.7%), influenza A (283, 37.7%), and AdV (346, 37.0%) had the greatest proportion of ED associated sampling, whilst single positive HBoV (65, 18.9%), HRV (1057, 17.3%), and HCoV 229E/NL63 (64, 15.46%) had the least (Table 2).

**Table 2:** Single positive sample characteristics by virus.

		Any Virus Positive	Rhinovirus A/B/C	Respiratory Syncytial Virus B	Respiratory Syncytial Virus A	Human Metapneumovirus	Adenovirus	Parainfluenza Virus 3	Influenza A	Enterovirus	Coronavirus OC43	Coronavirus 229E/NL63	Influenza B	Bocavirus (1–4)	Parainfluenza Virus 1	Parainfluenza Virus 4	Parainfluenza Virus 2
	**Count (% Positive)**	15082	6125	1394	1290	972	934	864	750	522	478	414	359	344	303	214	119
		(100)	(40.6)	(9.2)	(8.6)	(6.4)	(6.2)	(5.7)	(5.0)	(3.5)	(3.2)	(2.7)	(2.4)	(2.3)	(2.0)	(1.4)	(0.8)
**Sex**	**Male (%)**	8585	3608	763	678	535	521	496	408	299	280	250	192	193	168	123	71
		(56.9)	(58.9)	(54.7)	(52.6)	(55.0)	(55.8)	(57.4)	(54.4)	(57.3)	(58.6)	(60.4)	(53.5)	(56.1)	(55.4)	(57.5)	(59.7)
**Age (months)**	**Median (IQR)**	15	14	5	6	18	30	13	47	17	15	26	70	16	23	16	28
		(4–45)	(4–45)	(2–18)	(2–19)	(6–43)	(14–56)	(4–33)	(19–84)	(5–45)	(3–53)	(7–69)	(39–107)	(9–28)	(7–53)	(4–48)	(7–56)
**Age Group**	**<6 months**	4287	1764	698	617	219	54	251	81	137	148	96	19	46	66	62	29
		(28.4)	(28.8)	(50.1)	(47.8)	(22.5)	(5.8)	(29.1)	(10.8)	(26.2)	(31.0)	(23.2)	(5.3)	(13.4)	(21.8)	(29.0)	(24.4)
	**6–11 months**	2296	1056	206	197	156	130	158	50	64	54	48	23	74	33	36	11
		(15.2)	(17.2)	(14.8)	(15.3)	(16.0)	(13.9)	(18.3)	(6.7)	(12.3)	(11.3)	(11.6)	(6.4)	(21.5)	(10.9)	(16.8)	(9.2)
	**1–4 years**	5623	2100	369	385	432	542	342	316	227	170	152	111	204	146	76	51
		(37.3)	(34.3)	(26.5)	(29.8)	(44.4)	(58.0)	(39.6)	(42.1)	(43.5)	(35.6)	(36.7)	(30.9)	(59.3)	(48.2)	(35.5)	(42.9)
	**5–9 years**	1845	708	91	60	95	166	71	204	62	69	76	133	15	46	31	18
		(12.2)	(11.6)	(6.5)	(4.7)	(9.8)	(17.8)	(8.2)	(27.2)	(11.9)	(14.4)	(18.4)	(37.0)	(4.4)	(15.2)	(14.5)	(15.1)
	**10–15 years**	1031	497	30	31	70	42	42	99	32	37	42	73	5	12	9	10
		(6.8)	(8.1)	(2.2)	(2.4)	(7.2)	(4.5)	(4.9)	(13.2)	(6.1)	(7.7)	(10.1)	(20.3)	(1.5)	(4.0)	(4.2)	(8.4)
**Discharge Location**	**Inpatient**	10717	4643	985	948	655	559	552	435	373	355	320	199	265	200	146	82
		(71.1)	(75.8)	(70.7)	(73.5)	(67.4)	(59.9)	(63.9)	(58.0)	(71.5)	(74.3)	(77.3)	(55.4)	(77.0)	(66.0)	(68.2)	(68.9)
	**Emergency Department**	3573	1057	366	305	278	346	272	283	135	93	64	146	65	78	56	29
		(23.7)	(17.3)	(26.3)	(23.6)	(28.6)	(37.0)	(31.5)	(37.7)	(25.9)	(19.5)	(15.5)	(40.7)	(18.9)	(25.7)	(26.2)	(24.4)
	**Outpatient or Other Location**	792	425	43	37	39	29	40	32	14	30	30	14	14	25	12	8
		(5.3)	(6.9)	(3.1)	(2.9)	(4.0)	(3.1)	(4.6)	(4.3)	(2.7)	(6.3)	(7.2)	(3.9)	(4.1)	(8.3)	(5.6)	(6.7)
**^Length of Stay (days)**	**Median (IQR)**	2	3	2	2	2	2	2	1	2	3	3	1	3	2	2	2
		(1–6)	(1–13)	(1–5)	(1–4)	(1–4)	(0–3)	(1–6)	(0–3)	(1–3)	(1–12)	(1–14)	(0–3)	(1–9)	(1–4)	(1–8)	(1–7)
**Time to Sampling (days)**	**Median (IQR)**	1	1	0	1	0	1	1	1	1	1	1	1	1	1	1	1
		(0–2)	(0–3)	(0–1)	(0–1)	(0–1)	(0–1)	(0–1)	(0–1)	(0–1)	(0–3)	(0–3)	(0–1)	(0–2)	(0–1)	(0–2)	(0–2)
^**$**^ **Respiratory Tract Sampling Location**	**Upper Tract**	14601	5886	1369	1268	950	904	837	733	510	449	403	352	334	292	203	111
		(96.8)	(96.1)	(98.2)	(98.3)	(97.7)	(96.8)	(96.9)	(97.7)	(97.7)	(93.9)	(97.3)	(98.1)	(97.1)	(96.4)	(94.9)	(93.3)
	**Lower Tract**	418	220	23	17	16	23	23	13	9	27	8	6	10	8	10	5
		(2.8)	(3.6)	(1.6)	(1.3)	(1.6)	(2.5)	(2.7)	(1.7)	(1.7)	(5.6)	(1.9)	(1.7)	(2.9)	(2.6)	(4.7)	(4.2)
	**Other**	63	19	2	5	6	7	4	4	3	2	3	1	-	3	1	3
		(0.4)	(0.3)	(0.1)	(0.4)	(0.6)	(0.7)	(0.5)	(0.5)	(0.6)	(0.4)	(0.7)	(0.3)		(1.0)	(0.5)	(2.5)

Table Footnotes: Demographic and clinical characteristics of single virus positive detected. Total frequency and percentage of column total (n (%)) shown unless otherwise stated.

^Length of stay was calculated for inpatients only.

$Respiratory tract sampling location was grouped according to where in the respiratory tract the tested sample originated. Upper tract samples included nasopharyngeal aspirates, nasopharyngeal swabs, throat swabs, and combined nasopharyngeal/throat swabs. Lower tract samples included lung aspirates, bronchoalveolar lavages, endotracheal aspirates, bronchial brushing, and sputum samples. Samples were only included in the ’other’ category when entered as such into the pathology collection system by the referring clinician. #Includes samples positive for either coronavirus 229E or coronavirus NL63 over the full study period. Results for each assay is available in S1 Table.

Abbreviations: IQR: inter quartile range, N/A: Not Applicable

### Virus seasonality

**Fig 1:**
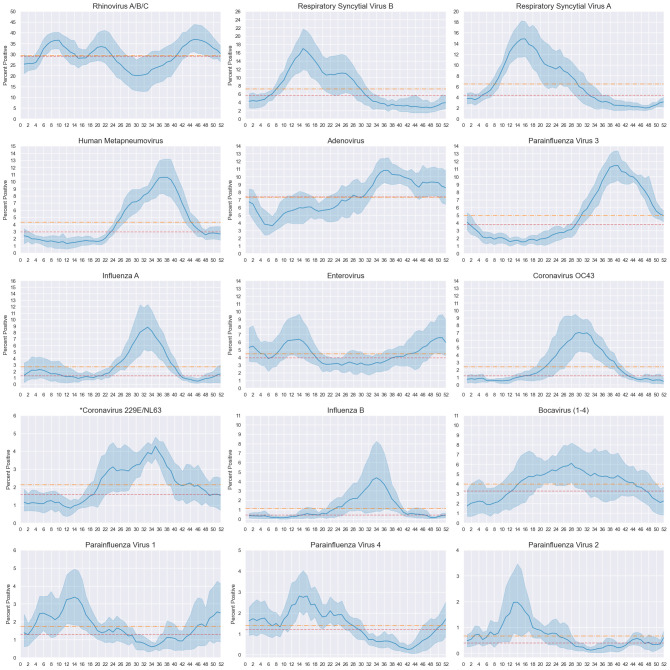
Average annual seasonal curve by virus: Test percent positive, five weekly moving average. Five weekly moving average test percent positive seasonal curves by virus across the study period (2014–2019). Mean percentage of positive samples (solid blue line) banded by 95% confidence interval (light blue) by epidemiological week. Measures of average test percent positive for each virus were calculated over the full study period, including mean (orange horizontal dashed and dotted lines) and median (red horizontal dashed lines). Percent positive axis scaled to each virus as to not diminish visibility of fluctuations for less frequently detected viruses. Viruses ordered from most (rhinovirus A/B/C) to least (parainfluenza 4) frequently detected. *Includes samples positive for either coronavirus 229E and/or coronavirus NL63 over the full study period. Results for each assay is available in S3 Fig.

Rhinovirus was consistently detected at high levels year-round (average yearly mean and median test percent positive 29%). Enterovirus was also consistently detected throughout the year, although at much lower levels (mean and median = 4%). Adenovirus and HBoV appear to show wide annual, seasonal curves, with AdV peaking late in the year (10.8%, week 36) and HBoV mid-year (6.1%, week 28). Clear seasonal peaks were visible for all other viruses (Fig 1).

Large bimodal peaks are seen in average seasonal curves for RSV-A and RSV-B, which both first peaked at weeks 16 and 15 respectively with a secondary peak occurring approximately 10 weeks afterwards. Several viruses had overlapping seasonality and peaked later in the year including HCoV OC43 (week 30), influenza A (week 33) and B (week 34), HCoV 229E/NL63 (week 35), HMPV (week 38), and hPIV3 (week 40). Whilst hPIV1, 2, and 4 had small peaks early in the year, peaking at week 14 for hPIV1 and 2, and week 16 for hPIV4 (Fig 1).

For influenza B and hPIV2, wide confidence intervals around their average annual seasonal peaks suggested variability between years, and a biennial pattern can be distinguished between each year of the study upon inspection of seasonal curves by epidemiological week and year. Influenza B and hPIV2 appear to have larger peaks on odd years (2015, 2017, 2019), with the difference more exaggerated for influenza B (S3 Fig).

### Strength of seasonality

Viruses with the lowest strength of seasonality were HRV (annual positivity range 0.88) and EV (0.73). Adenovirus and HBoV had a higher annual seasonal strength (annual positivity ranges of 2.05 and 2.41 respectively) but were below the threshold positivity range of 2.5 and were also designated to have annual seasonality of low strength. All other viruses displayed at least moderate annual seasonal strength (positivity range of ≥2.5; Table 3).

**Table 3:** Strength of seasonality by virus.

		Rhinovirus A/B/C	Respiratory Syncytial Virus B	Respiratory Syncytial Virus A	Human Metapneumovirus	Adenovirus	Parainfluenza Virus 3	*Influenza A	Enterovirus	Coronavirus OC43	Coronavirus 229E/NL63	*Influenza B	Bocavirus (1–4)	Parainfluenza Virus 1	Parainfluenza Virus 4	Parainfluenza Virus 2
**Positivity Range**	**Annual**	0.88	2.83	2.92	4.06	2.05	3.66	5.63	0.73	4.92	3.02	5.98	2.41	2.50	2.69	3.39
	**Biennial Even Years**	1.25	2.78	3.15	4.29	2.40	3.98	5.70	1.59	5.68	3.49	3.68	2.78	3.26	2.65	2.41
	**Biennial Odd Years**	0.78	2.97	3.11	3.92	1.85	3.59	5.71	1.12	4.95	3.13	6.77	2.37	3.49	2.42	4.80
**Biennial Difference**		0.47	0.19	0.04	0.37	0.55	0.40	0.01	0.47	0.73	0.36	3.09	0.41	0.24	0.23	2.39
**Final Seasonality**		Annual	Annual	Annual	Annual	Annual	Annual	Annual	Annual	Annual	Annual	Biennial	Annual	Annual	Annual	Biennial
**Final Seasonal Strength**		Low	Moderate	Moderate	Moderate	Low	Moderate	High	Low	Moderate	Moderate	High	Low	Moderate	Moderate	Moderate

Table Footnotes: Viruses with a seasonal positivity range of <2.5 were considered to have low seasonality, 2.5-<5 were considered to have moderate seasonality, and ≥5 was considered to have high seasonality. A 13 week moving average was used to smooth counts of positive detections by virus. Weekly positive counts were standardised across each year of the study period and the proportion that each weekly count contributed to the total yearly count was calculated. The range between highest and lowest smoothed weekly count was used to determine the seasonal strength of each virus. Seasonal strength was calculated annually and biennially (both for even and odd years).

*Results calculated using 2014–2018 results only. Due to an unexpectedly high number of influenza cases in 2019, a shift in testing behaviour for suspected influenza patients occurred at the Children’s Hospital at Westmead. Due to an unseasonably early and large influenza season experienced in 2019 [18], children were tested using a Cepheid GeneXpert influenza rapid test and if positive were not usually tested again using the full multiplex assay. As such there was likely an underestimation of influenza cases during 2019 using multiplex results alone.

High seasonal strength was observed for influenza A across all comparisons, with high annual seasonal strength (positivity range 5.6). Difference in positivity ranges between biennial even and odd years (0.01) was the smallest among all viruses. Low biennial seasonal differences were also observed for RSV-A (0.04) and RSV-B (0.19), which both had moderate annual seasonality (positivity ranges of 2.92 and 2.83, respectively; Table 3).

Coronavirus OC43 was calculated to have high biennial seasonal strength when calculated across even years (2014, 2016, 2018; positivity range 5.68), but had positivity ranges approaching the cut-off for high seasonality for annual (4.92) and biennial (odd years; 4.95) seasonality. However, as this was below the threshold for high seasonal strength and was designated to have moderate annual seasonality. Biennial seasonality was observed in influenza B and hPIV2. Influenza B had the largest difference in biennial seasonal strengths (3.09), with higher seasonal strength observed for odd years (6.77). Biennial seasonality occurring in odd years was also observed for hPIV2 (difference in biennial seasonal strengths 2.39), although with moderate seasonal strength (4.80; Table 3).

### Viral co-detections

**Fig 2:**
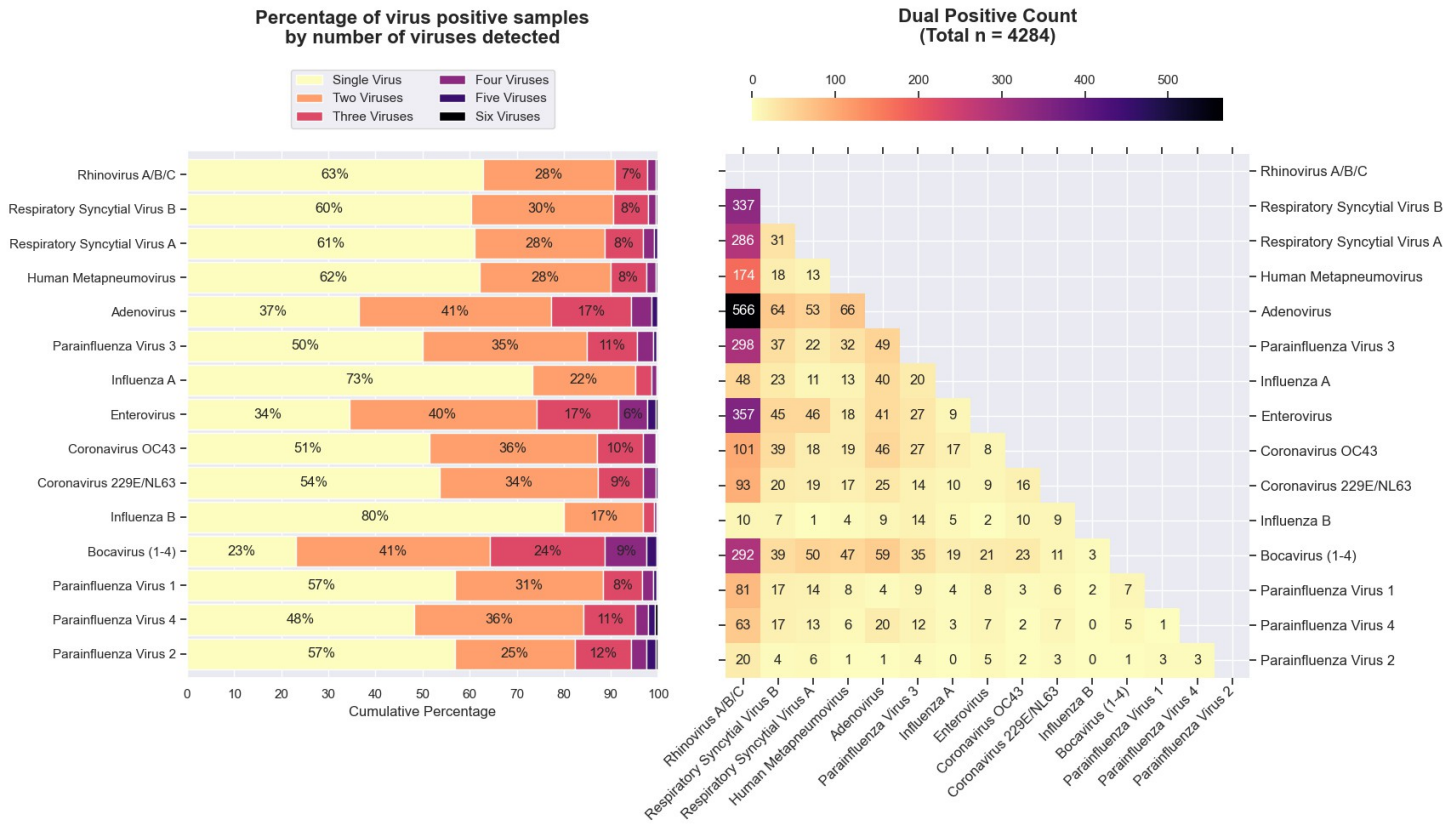
Multi-positive samples by virus, with dual positive heatmap. Bar chart representing the number of viruses detected within samples by virus.

Multi-virus detections (co-detections) represented 26.5% (5428) of all positive samples. Most were dual positive detections (4284/5428, 78.9%), and from children aged 1–4 years (2560, 47.2%). Children aged 6–11 months experienced the highest proportion of multi-virus detections (1287/3583, 35.9%), followed closely by children aged 1–4 years (2553/8183, 31.2%), whilst 10–15 year-olds had the lowest (114/1145, 10.0%; S2 Table). Influenza was least likely to be co-detected with other viruses; 80% of influenza B and 73% of influenza detections were from single positive samples. However several viruses were frequently associated with multi-positive samples, with a minority only 23% of HBoV, 34% of EV, and 37% of AdV detections being from single positive samples (Fig 2).

Among dual positive samples (4284), HRV (2726, 63.6%) and AdV (1043, 24.3%) were most frequently detected, whilst hPIV2 (53, 1.2%) and influenza B (76, 1.8%) were least frequently detected (Fig 2). The most common dual virus detection pairing was AdV-HRV (566, 13.2%), followed by EV-HRV (357, 8.3%), and RSV-B-HRV (337, 7.9%; Figs 2 and S4). Proportionally, HRV was the most common virus to appear across all dual positive pairings, with exception of influenza B, where 18.4% of influenza B dual detections were with hPIV3 (Fig 2). There was a total of 404 unique combinations of multi-virus detections. Rhinovirus appears in all top ten instances, and HRV, AdV, HBoV, and EV are implicated in 41 of the top 50 multi-virus detections. Of the nine remaining combinations, the first non-HRV, AdV, HBoV, EV combination appears at 28^th^ most common (RSV-B-HCoV OC43, n = 39). The only quadruple detection included in the top 50 combinations was HRV-AdV-HBoV-EV (22), which was the 48^th^ most common multi-detection combination (S4 Fig).

## Discussion

Among this large paediatric tertiary hospital population, most respiratory virus detections, both single and multi-virus, occurred in children aged 1–4 years. There was also a male predominance, a not well understood phenomenon observed in other studies of paediatric ARI [19–22]. Most samples were taken from the upper respiratory tract, which is expected and is standard diagnostic practice for uncomplicated ARI.

### HRV, RSV, HMPV, and hPIV3 are key contributors to paediatric ARI

Viruses were detected in approximately two-thirds of samples overall, and the proportion increased with younger age. This is consistent with other paediatric studies of viral ARI [20, 22, 23]. Rhinovirus was the most frequently detected virus and was observed to have low seasonal strength and occur year-round. Several other studies similarly found that picornaviruses (joint EV/HRV detection) were the most common ARI-associated viruses among children [22, 23], with one study finding that children were infected with HRV/EV at two and a half times that of adults [23]. Although often overlooked in terms of its contribution to clinically significant disease, HRV is frequently associated with bronchiolitis and LRTI in children [19, 24], though the severity of HRV in comparison to other viral causes of LRTI is uncertain. When detected alone HRV has been associated with an increased severity of LRTI [19], although there was no difference found among children with bronchiolitis [24]. There are also studies implicating HRV infection during infancy, particularly HRV-C, with increased risk of recurrent wheeze by three and four years of age [25, 26]. There are three HRV species which include a broad range of serotypes and genotypes, and although evaluated here as a single virus detection, considerable heterogeneity likely underly our observations. The opacity of current HRV testing to serotype specific epidemiology and clinical clustering is a key limitation. Inclusion of subtype testing may assist in improving our understanding which HRV infections result in higher severity of HRV associated ARI and risk of chronic respiratory conditions.

Enterovirus was similarly observed to have low seasonal strength with no clear annual epidemics. Over the course of the study EV was persistently detected at lower levels and observed to have sporadic outbreaks. Similar findings have been seen in the United States which revealed a flat seasonal profile across southern states, which are most comparable the climate in Sydney [27]. From previous studies we know that there are a high number of EV-A serotypes which circulate among children presenting to our hospital that are detected by our current assay [28], and it can be difficult to identify which detections may be the most clinically relevant in diagnosis and treatment of paediatric ARI. There is growing evidence of EV-D68 role in severe ARI, including a study using samples from children presenting to our hospital [29, 30]. Larsson and colleagues also found that most presentation of infection with another enterovirus, coxsackievirus A10 (CV-A10), were associated with ARI particularly among children under the age of five [30]. Similarly to HRV, whilst it may be considered unfeasible to test for EV down to serotype for all infections, consideration should be given to testing for select serotypes, such as EV-D68 and CV-A10, in molecular testing for ARI surveillance.

Bronchiolitis and LRTI in young children are most often associated with RSV infection [8, 9, 11, 19, 24] and this is reflected among children in our study. We found RSV-B and RSV-A were second and third most frequently detected after HRV, despite not being persistently detected year-round, similar to observations from other studies [22, 23]. Detections were associated most frequently in the youngest children, with around half in children aged less than six months. Both subtypes were observed to have moderate annual seasonal patterns with bimodal peaks in autumn and winter, more exaggerated in RSV-B. Progression of targeted antiviral therapies and RSV maternal and childhood immunisation [31–34], highlight the need for a solid understanding of the seasonal patterns of infection of both subtypes to maximise effectiveness of these preventatives. Year-round surveillance is necessary to alert of any unusual seasonal changes, particularly considering the disruption of seasonal patterns associated with non-pharmaceutical interventions employed during the COVID-19 pandemic [8, 13, 35].

Human metapneumovirus and hPIV3 are additional important contributors to paediatric ARI and have a considerable global burden [10, 36]. We observed HMPV and hPIV3 to be the fourth and sixth most frequent independently detected viruses in this study. Similarities in the frequencies of detection of these two viruses were observed in other studies [20, 22, 23]. Both HMPV and hPIV3 had moderate seasonal strength, with overlapping spring epidemics which peaked on average only two weeks apart. This substantial overlap of seasonality may be importance with respect to future preventative interventions, such as vaccination. A recent phase one clinical trial of a joint HMPV-hPIV3 vaccine revealed above baseline neutralising antibody titres a year post immunisation for HMPV, although found that this waned more quickly for hPIV3 [37]. Despite a possible shorter effective period for such vaccinations, well-timed and targeted programs could have the potential to alleviate the burden among children most at-risk of severe infection.

### Influenzas and parainfluenzas

As expected, influenza showed seasonal winter epidemics with high seasonal strength. This seasonality was observed on an annual basis for influenza A whilst for influenza B, although detected to increase each year, had a clear odd-year biennial cycle. One Japanese study found a similar biennial pattern of influenza seroprevalence among children, driven by influenza B, and noted that this was not seen in adults [38]. Proportions of influenza detection in our study were comparable to other studies with a similar breadth of included viruses [22, 23]. Influenza A and B were both noted to have the highest proportion of single positive samples among those in this study; 73% and 80% respectively. This low rate of co-detection was noted in other studies [23, 39] and may be explained by influenzas fast replication and competitive interference with other viruses [40].

Although half of hPIV associated LRTI in children under five years is attributable to hPIV3 [10], all four serotypes of hPIV can cause significant lower tract disease [36, 41, 42], highlighting the need to better understand their epidemiology. Whilst hPIV types 1, 2, and 4 were among the least frequently detected viruses in our study, the large number of samples over the study period enabled us to get a clearer picture of their seasonal patterns. We found that all three serotypes peak in autumn, which contrasts with hPIV3 which peaked much later in the year in spring. We observed a biennial cycle for hPIV2, which was confirmed by seasonal strength analyses, a pattern observed in other studies [41, 42]. Visual inspection of hPIV1 over the full study period indicated that there may be a biennial seasonal pattern with peaks occurring early in even years. However, upon analysis of seasonal strength this was not borne out. This could be due to the high variability of seasonal onset which occurred early in the year in 2014 and 2016, however detection began to rise earlier for the following cycles, where detections rose in late 2017 and late 2019. Biennial seasonality for hPIV1 was seen in one Perth study [41], whilst a study from Korea found hPIV1 was detected sporadically year-round [42]. As hPIV1 is estimated to be attributable for over a quarter of instances of hPIV-associated LRTI globally [10], improvements in understanding its seasonality are essential.

### Low seasonal strength of AdV, and HBoV

Adenovirus and HBoV were also observed to have low annual seasonal strength, though more so than that of HRV and EV, with the AdV seasonal curve peaking in autumn, and HBoV peaking in late winter. Similar findings of virus seasonality were seen for AdV [22] and HBoV [43] in other studies. We observed that both AdV and HBoV have very high rates of co-detection in comparison to other viruses, with single detections in only 37% and 23% of their total detections. Other studies reported similarly high rates of co-detection among AdV positive samples, ranging from 49% to 65% [20, 23, 39, 44]. Fewer studies included HBoV in their testing panels however, of those that did, between 49% and 92% HBoV positive samples were multi-virus positive [19, 22]. The dual detection of AdV and HRV represented 10% of multi-virus detections in our study. This co-detection was also most [20, 44] or second most common in other studies [22, 23]. Testing for AdV and HBoV are cross-subtype which, like HRV and EV, which further complicates evaluation of their contribution to paediatric ARI as it is unclear which detections are clinically relevant.

### Co-detection of viruses in multiplex assays

With increasing use of multiplex assays with improved assay sensitivity available for clinical diagnosis there are increasing instances of co-detection of viruses. We observed that over a quarter of positive samples in our dataset (26.5%) have two or more viruses detected. This is slightly higher than other similar studies which report co-detection rates from 6.4% to 21.7% of positive tests [20–23, 39]. Studies with narrower focus on younger children with more severe disease reported co-detection rates as high as 56.2% [19, 24], however this disparity could be explained by the increased number of co-detections observed in children under 5 years of age [23, 39, 44, 45]. We observed that there was an increased density of infection among children aged 1–4 years of age; which was most evident as the number of viruses co-detected increased. This non-linear pattern of infection was also observed by Martin and colleagues, who found that children aged 6–24 months were most frequently multi-virus positive [20]. This pattern could be explained by the increased mobility of this age group, with childcare attendance associated with increased risk of ARI infection for both the child and other household members [46].

There is growing interest into how multi-positive infections differ in their clinical manifestations to single positive diagnoses. However, there are inconsistent findings around how multi-virus infections impact disease severity and burden [47]. Martin and colleagues found that children admitted with single virus associated ARI were more likely to be hospitalised, require supplemental oxygen, have extended lengths of stay, and require admission to intensive care than in instances of multi-virus associated infections [20]. In contrast, several studies have noted an increased disease severity in young children with LRTI with co-detection of HRV and RSV [19, 24]. This combination of co-detection occurred frequently in our study, with RSV-B/HRV dual detections being the third most common multi-virus detection, and RSV-A/HRV coming in sixth. The high frequency of these dual-positive detections provides impetus and opportunity for further investigation of the clinical significance of virus-virus co-detection.

### Study strengths and limitations

Strengths of this study include the large sample size from a large tertiary paediatric hospital which services a wide geographic area of Australia’s most populous state. Additionally, the study duration enabled seasonal analyses to be made over a six-year period, providing three seasons of reference for biennial viruses, and six for annual viruses. The year-round routine use of multiplex PCR at the Children’s Hospital at Westmead allows comparison of a wide range of viruses reducing bias to suspected cause by referring clinicians.

There are several limitations to this study. Firstly, this is a retrospective, observational study that uses routinely collected laboratory data beyond its intended purpose which is to support clinical management through identification of respiratory pathogens. We have assumed that children undergoing respiratory panel testing were experiencing ARI symptoms as the indication for clinician directed testing, although it is possible that some children presented with a broader set of clinical features, *i*.*e*. fever alone, or were tested for pre-operative screening purposes. Additionally, PCR by nature cannot distinguish between infectious and non-infectious viral particles, and in some instances viral nucleic acid can be found in patients long after clinical infection is resolved, an issue highlighted early in the COVID-19 pandemic as it was often tied to release from self-isolation and quarantine measures.

An unseasonably early and large influenza epidemic in 2019 saw the Children’s Hospital at Westmead make use of influenza-specific testing to reduce the volume of multiplex testing for the laboratory staff. This will have led to an underestimation of influenza detections for that year using multiplex data alone. Due to this, 2019 was removed from seasonal strength analyses for influenza viruses, and there is a widening of the confidence intervals for influenza viruses in the average epidemiological curves. We believe this will have had negligible impact on the co-detection analyses, due to the low number of co-detections among influenza positive samples in the other years of this study.

The change in laboratory assay in 2018 may have had an impact on our findings. Although we did not find an effect on the test positive proportion for most viruses, there was a marked increase in test positive proportion of HRV, AdV, HBoV, and EV. Testing for these viruses covers many subtypes/serotypes and may have increased sensitivity in the AP26 assay compared to the RV15.

Finally, we have not accounted for seasonal overlap in our analyses of co-detection of multiple viruses. Due to this there may be instances where low co-detection rates may be primarily due to reduced seasonal overlap with other viruses rather than any other virological or biological interferences.

## Conclusion

To summarise, HRV, RSV, HMPV, and hPIV3 are the most frequently detected respiratory viruses in children presenting to this tertiary paediatric hospital, associated with over 70% of single detections and 85% of detections overall. Most respiratory viruses show some degree of seasonality with an annual, rather than biennial, seasonal cycle. Rates of multi-virus detection are high, particularly among younger children, and represent over a quarter of positive samples in this study indicating the need for continued evaluation of their clinical impact. Enhanced pan-pathogen surveillance that integrates both laboratory and clinical data could support the delivery effective public health responses, such as promoting vaccination in relation to seasonal patterns, and in healthcare activity surge preparedness.

## Supporting information

S1 Fig STROBE-style sample flow chart. Line listed test results for all respiratory samples tested at the Children’s Hospital at Westmead between 22/09/2013 and 30/06/2020 were extracted from hospital databases. ’Test’ refers to a specific individual virus test within the multiplex PCR assay (i.e. adenovirus or bocavirus), whilst ’sample’ refers to an individual respiratory sample taken during admission or on presentation to the Children’s Hospital at Westmead. For example, one sample could have 15 test results (one for each virus tested) attached to it. Only respiratory samples from children aged under 16 years of age that underwent complete PCR testing were included in the final dataset. # Only most recent valid/corrected result taken forward for analysis. *Incomplete year of sampling. ^Significant changes to testing strategy employed due to the COVID-19 pandemic.
(TIF)

S2 Fig Geographic distribution of sampling (NSW only). The residential postcode of children who underwent PCR testing of a respiratory sample taken at the Children’s Hospital at Westmead show a similar distribution to the New South Wales (NSW) population. Only postcodes within NSW were plotted, as this represents over 95.5% of samples in the analysed dataset. Inset image of Greater Sydney region for additional resolution. Location of the Children’s Hospital at Westmead indicated by the green cross (’x’) on both main and inset maps. Higher counts indicated by darker colour. Postcodes designated as within greater state capital areas as per Australian Bureau of Statistics (ABS) Greater Capital City Statistical Areas (GCCSA) 2016.
(TIF)

S3 Fig Seasonal curve by virus and assay (2014–2019): Test percent positive, five weekly moving average. Five weekly moving average test percent positive seasonal curves by virus across the study period by epidemiological week. Approximate calendar months (January, March, June, September) indicated on the x-axis for ease of reading; these equate to epidemiological weeks 1, 13, 26, and 39 for each year (2014–2019). Measures of average test percent positive for each virus were calculated over the full study period, including mean (orange horizontal dashed and dotted lines) and median (red horizontal dashed lines). During epidemiological week 10 of 2018 there was a change in assay used at the Children’s Hospital at Westmead from Seegene Seeplex Respiratory Virus 15 (solid blue line) to Seegene Allplex Respiratory 26 (solid orange line). Percent positive axis scaled to each virus as to not diminish visibility of fluctuations for less frequently detected viruses. Viruses ordered from most (rhinovirus A/B/C) to least (Coronavirus NL63 (AP26)) frequently detected. Due to this assay change, a combined percentage positive was calculated for coronavirus 229E and NL63 across the full study. Coronaviruses 229E and NL63 were detected on a single channel in the older assay, whereas the new assay distinguishes between the two. Individual assay curves (last two in figure) were also plotted for comparison. For these coronaviruses, measures of average were calculated only for the time periods of assay use to avoid underestimation caused by period of ’zero detections’.
(TIF)

S4 Fig Multi-positive co-detections UpSet plot. Total number of co-detections of two or more viruses within a single sample. Multi-virus detections occurred in 5421 samples (26.4% of positive samples). A minimum cut-off of 20 detections was used, which represents the top 50 combinations of co-detections. Dual detections are shown in dark blue, triple detections in green, and four-virus detections in red. Quintuple virus co-detections not shown due to largest combination subgroup containing only five samples.
(TIF)

S1 Table Single positive sample characteristics of coronaviruses 229E and NL63. Table Footnotes: In March 2018 there was a change in assay used at the Children’s Hospital at Westmead from Seegene Seeplex Respiratory Virus 15 (RV15) to Seegene Allplex Respiratory 26 (AP26). Coronaviruses 229E and NL63 were detected on a single channel in the older assay, whereas the new assay distinguishes between the two. *Coronavirus 229E/NL63 (RV15) refers to detections tested using the Seegene Seeplex Respiratory Virus 15 PCR assay between January 2014 and March 2018. #Individual results for coronavirus 229E and coronavirus NL63 were available for samples tested using the Seegene Allplex Respiratory 26 (AP26) assay between March 2018 and December 2019. Total frequency and percentage of column total (n (%)) shown unless otherwise stated. ^Length of stay was calculated for inpatients only. $Respiratory tract sampling location was grouped according to where in the respiratory tract the tested sample originated. Upper tract samples included nasopharyngeal aspirates, nasopharyngeal swabs, throat swabs, and combined nasopharyngeal/throat swabs. Lower tract samples included lung aspirates, bronchoalveolar lavages, endotracheal aspirates, bronchial brushing, and sputum samples. ‘Other’ samples included respiratory samples without definite categorisation. Abbreviations: IQR: inter quartile range, N/A: Not Applicable.
(DOCX)

S2 Table Co-detection characteristics. Table Footnotes: Demographic and clinical characteristics of positive samples by number of viruses detected. Total frequency and percentage of column total (n (%)) shown unless otherwise stated. *IRSD is a general socio-economic score providing insight into socio-economic disadvantage. Those in the lowest quintile represent individuals from areas which many individuals have low access to resources and reduced abilities to participate in society. ^Length of stay was calculated for inpatients only. $Respiratory tract sampling location was grouped according to where in the respiratory tract the tested sample originated. Upper tract samples included nasopharyngeal aspirates, nasopharyngeal swabs, throat swabs, and combined nasopharyngeal/throat swabs. Lower tract samples included lung aspirates, bronchoalveolar lavages, endotracheal aspirates, bronchial brushing, and sputum samples. ‘Other’ samples included respiratory samples without definite categorisation. Abbreviations: IQR: inter quartile range, IRSD: index of relative socio-economic disadvantage, N/A: Not Applicable.
(DOCX)
