# Supplementary figures and images for: Respiratory virus detections in children presenting to an Australian paediatric referral hospital pre-COVID-19 pandemic, January 2014 to December 2019
Source: PLoS One. 2025 Jan 22;20(1):e0313504. doi: 10.1371/journal.pone.0313504 (PMC12140113; doi:10.1371/journal.pone.0313504)

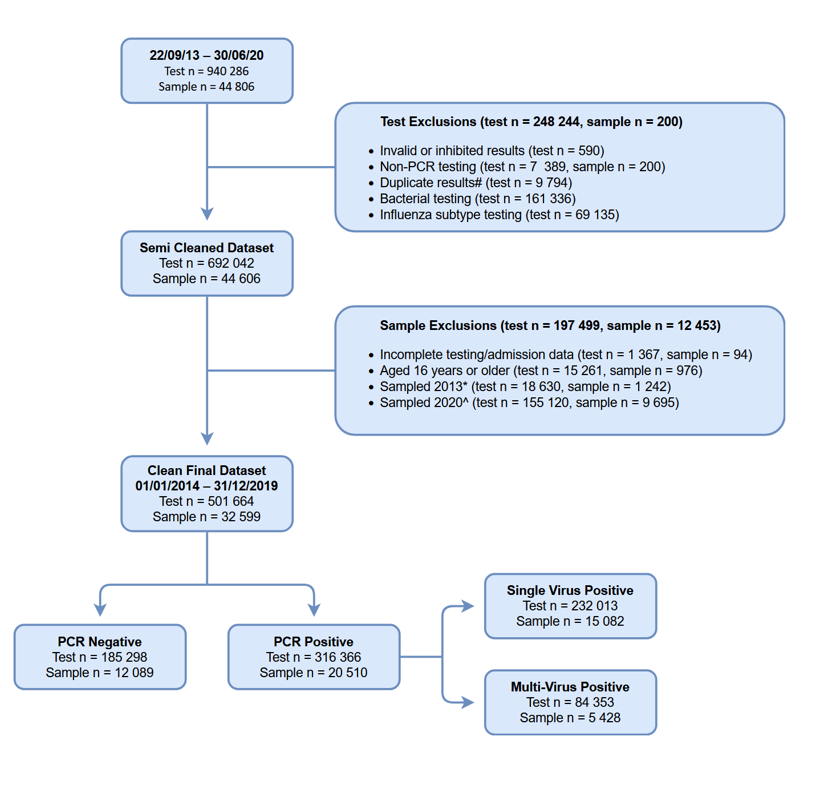

Supplement: S1 Fig — Line listed test results for all respiratory samples tested at the Children’s Hospital at Westmead between 22/09/2013 and 30/06/2020 were extracted from hospital databases. ’Test’ refers to a specific individual virus test within the multiplex PCR assay (i.e. adenovirus or bocavirus), whilst ’sample’ refers to an individual respiratory sample taken during admission or on presentation to the Children’s Hospital at Westmead. For example, one sample could have 15 test results (one for each virus tested) attached to it. Only respiratory samples from children aged under 16 years of age that underwent complete PCR testing were included in the final dataset. # Only most recent valid/corrected result taken forward for analysis. *Incomplete year of sampling. ^Significant changes to testing strategy employed due to the COVID-19 pandemic. (TIF) [file pone.0313504.s001.tif]

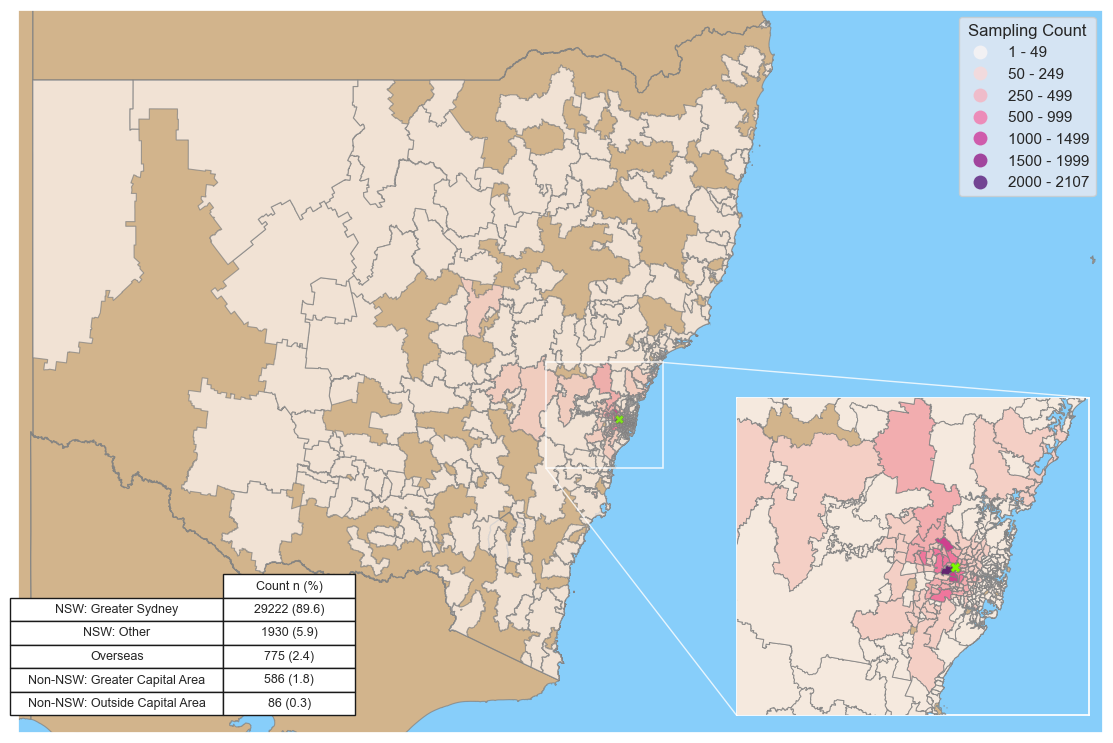

Supplement: S2 Fig — The residential postcode of children who underwent PCR testing of a respiratory sample taken at the Children’s Hospital at Westmead show a similar distribution to the New South Wales (NSW) population. Only postcodes within NSW were plotted, as this represents over 95.5% of samples in the analysed dataset. Inset image of Greater Sydney region for additional resolution. Location of the Children’s Hospital at Westmead indicated by the green cross (’x’) on both main and inset maps. Higher counts indicated by darker colour. Postcodes designated as within greater state capital areas as per Australian Bureau of Statistics (ABS) Greater Capital City Statistical Areas (GCCSA) 2016. (TIF) [file pone.0313504.s002.tif]

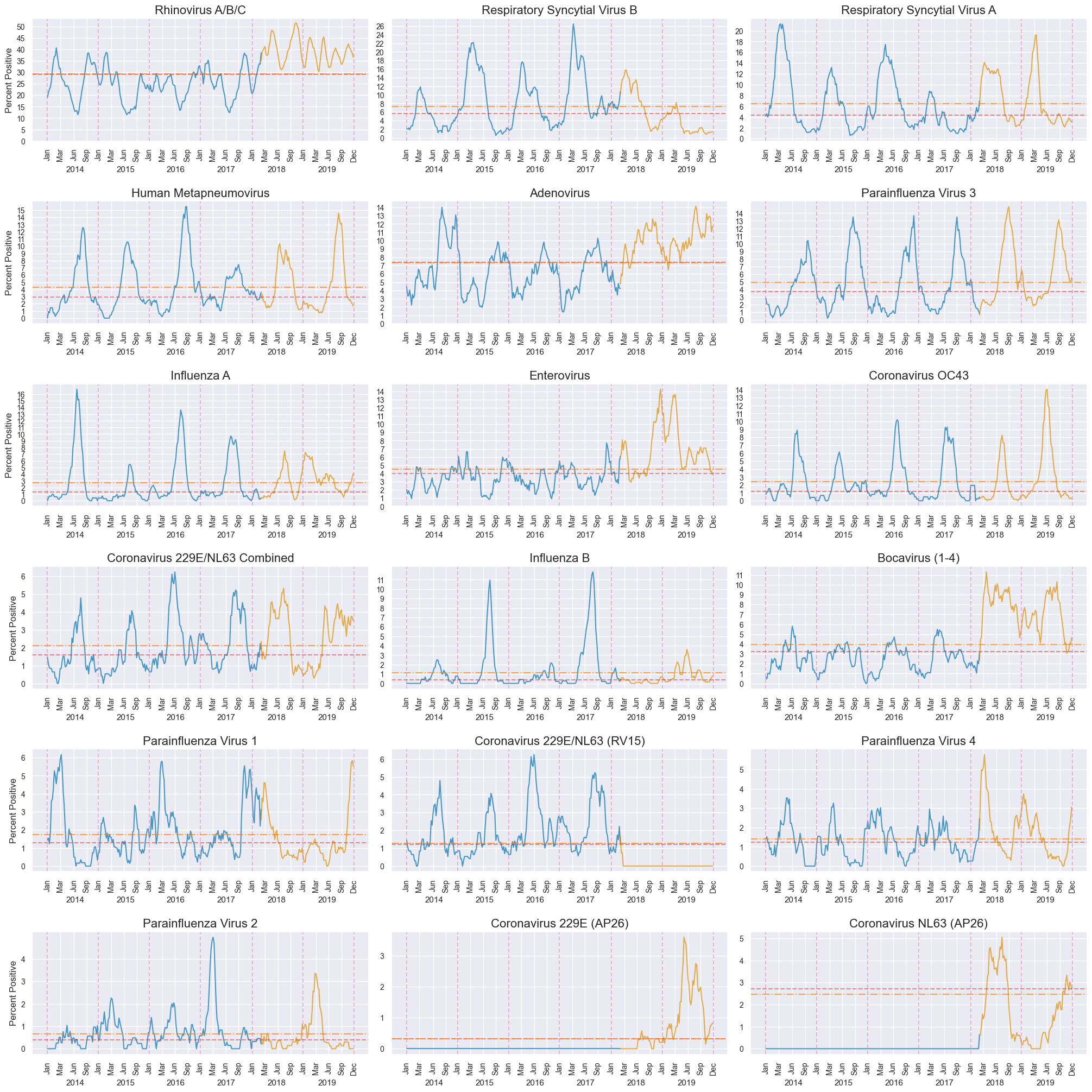

Supplement: S3 Fig — Five weekly moving average test percent positive seasonal curves by virus across the study period by epidemiological week. Approximate calendar months (January, March, June, September) indicated on the x-axis for ease of reading; these equate to epidemiological weeks 1, 13, 26, and 39 for each year (2014–2019). Measures of average test percent positive for each virus were calculated over the full study period, including mean (orange horizontal dashed and dotted lines) and median (red horizontal dashed lines). During epidemiological week 10 of 2018 there was a change in assay used at the Children’s Hospital at Westmead from Seegene Seeplex Respiratory Virus 15 (solid blue line) to Seegene Allplex Respiratory 26 (solid orange line). Percent positive axis scaled to each virus as to not diminish visibility of fluctuations for less frequently detected viruses. Viruses ordered from most (rhinovirus A/B/C) to least (Coronavirus NL63 (AP26)) frequently detected. Due to this assay change, a combined percentage positive was calculated for coronavirus 229E and NL63 across the full study. Coronaviruses 229E and NL63 were detected on a single channel in the older assay, whereas the new assay distinguishes between the two. Individual assay curves (last two in figure) were also plotted for comparison. For these coronaviruses, measures of average were calculated only for the time periods of assay use to avoid underestimation caused by period of ’zero detections’. (TIF) [file pone.0313504.s003.tif]

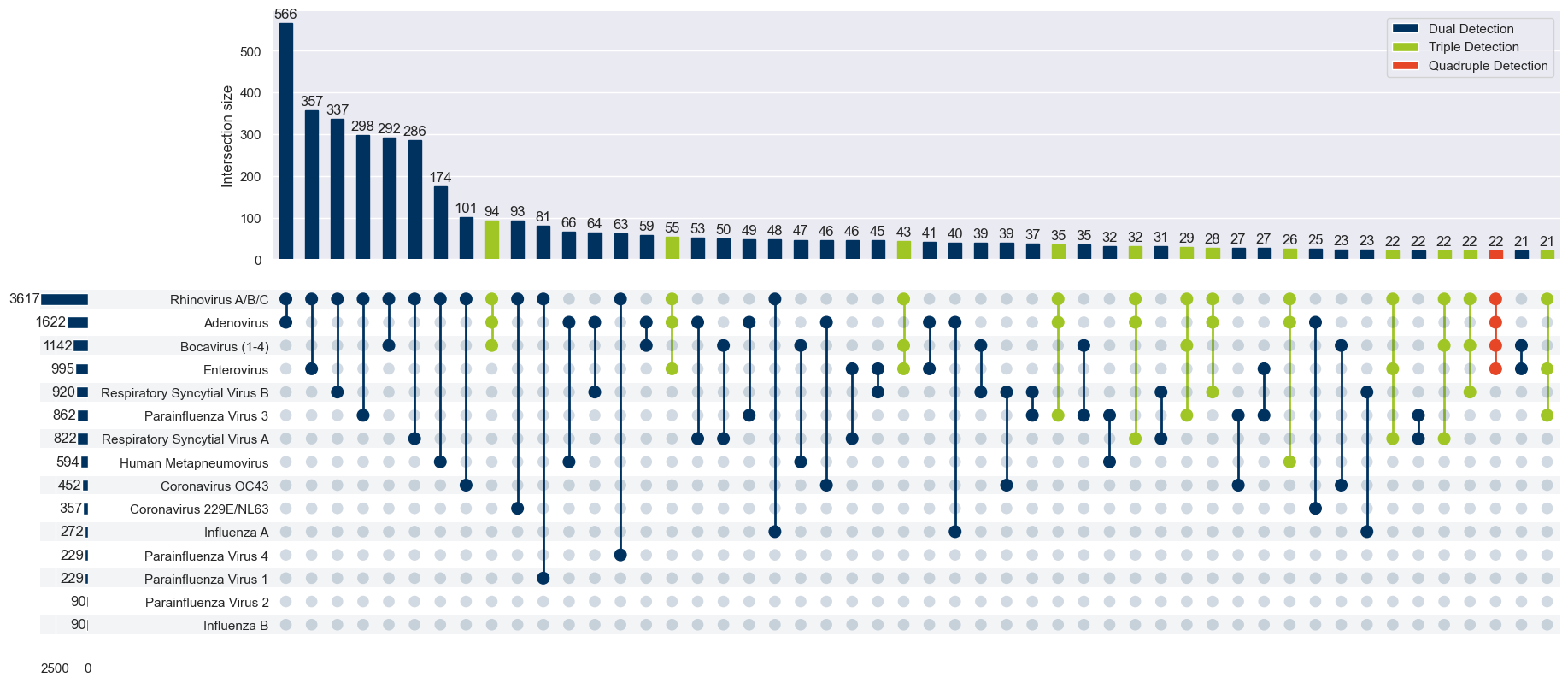

Supplement: S4 Fig — Total number of co-detections of two or more viruses within a single sample. Multi-virus detections occurred in 5421 samples (26.4% of positive samples). A minimum cut-off of 20 detections was used, which represents the top 50 combinations of co-detections. Dual detections are shown in dark blue, triple detections in green, and four-virus detections in red. Quintuple virus co-detections not shown due to largest combination subgroup containing only five samples. (TIF) [file pone.0313504.s004.tif]
