# Supplementary material for: Respiratory virus detections in children presenting to an Australian paediatric referral hospital pre-COVID-19 pandemic, January 2014 to December 2019
Source: PLoS One. 2025 Jan 22;20(1):e0313504. doi: 10.1371/journal.pone.0313504 (PMC12140113; doi:10.1371/journal.pone.0313504)
Supplement: S1 Table — Table Footnotes: In March 2018 there was a change in assay used at the Children’s Hospital at Westmead from Seegene Seeplex Respiratory Virus 15 (RV15) to Seegene Allplex Respiratory 26 (AP26). Coronaviruses 229E and NL63 were detected on a single channel in the older assay, whereas the new assay distinguishes between the two. *Coronavirus 229E/NL63 (RV15) refers to detections tested using the Seegene Seeplex Respiratory Virus 15 PCR assay between January 2014 and March 2018. #Individual results for coronavirus 229E and coronavirus NL63 were available for samples tested using the Seegene Allplex Respiratory 26 (AP26) assay between March 2018 and December 2019. Total frequency and percentage of column total (n (%)) shown unless otherwise stated. ^Length of stay was calculated for inpatients only. $Respiratory tract sampling location was grouped according to where in the respiratory tract the tested sample originated. Upper tract samples included nasopharyngeal aspirates, nasopharyngeal swabs, throat swabs, and combined nasopharyngeal/throat swabs. Lower tract samples included lung aspirates, bronchoalveolar lavages, endotracheal aspirates, bronchial brushing, and sputum samples. ‘Other’ samples included respiratory samples without definite categorisation. Abbreviations: IQR: inter quartile range, N/A: Not Applicable. (DOCX) [file pone.0313504.s005.docx]

|  |  | ***Coronavirus 229E/NL63 (RV15)** | **^#^Coronavirus 229E (AP26)** | **^#^Coronavirus NL63 (AP26)** |
| --- | --- | --- | --- | --- |
|  | **Count**  **(% Positive)** | 258 (1.7) | 46 (0.3) | 110 (0.7) |
| **Sex** | **Male (%)** | 151  (58.5) | 30  (65.2) | 69  (62.7) |
| **Age (months)** | **Median (IQR)** | 18 (7-57) | 54 (15-114) | 31 (6-78) |
| **Age Group** | **<6 months** | 59 (22.9) | 10 (21.7) | 27 (24.5) |
|  | **6-11 months** | 36 (14.0) | 1 (2.2) | 11 (10.0) |
|  | **1-4 years** | 102 (39.5) | 13 (28.3) | 37 (33.6) |
|  | **5-9 years** | 40 (15.5) | 17 (37.0) | 19 (17.3) |
|  | **10-15 years** | 21 (8.1) | 5 (10.9) | 16 (14.5) |
| **Discharge Location** | **Inpatient** | 205 (79.5) | 35 (76.1) | 80 (72.7) |
|  | **Emergency Department** | 34 (13.2) | 8 (17.4) | 22 (20.0) |
|  | **Outpatient or Other Location** | 19 (7.4) | 3 (6.5) | 8 (7.3) |
| **^Length of Stay (days)** | **Median (IQR)** | 3 (1-16) | 3 (1-13) | 3 (1-11) |
| **Time to Sampling (days)** | **Median (IQR)** | 1 (0-3) | 1 (0-3) | 1 (0-5) |
| **^$^Respiratory Tract Sampling Location** | **Upper Tract** | 250 (96.9) | 44 (95.7) | 109 (99.1) |
|  | **Lower Tract** | 6 (2.3) | 2 (4.3) | - |
|  | **Other** | 2 (0.8) | - | 1 (0.9) |
