# Supplementary material for: Respiratory virus detections in children presenting to an Australian paediatric referral hospital pre-COVID-19 pandemic, January 2014 to December 2019
Source: PLoS One. 2025 Jan 22;20(1):e0313504. doi: 10.1371/journal.pone.0313504 (PMC12140113; doi:10.1371/journal.pone.0313504)
Supplement: S2 Table — Table Footnotes: Demographic and clinical characteristics of positive samples by number of viruses detected. Total frequency and percentage of column total (n (%)) shown unless otherwise stated. *IRSD is a general socio-economic score providing insight into socio-economic disadvantage. Those in the lowest quintile represent individuals from areas which many individuals have low access to resources and reduced abilities to participate in society. ^Length of stay was calculated for inpatients only. $Respiratory tract sampling location was grouped according to where in the respiratory tract the tested sample originated. Upper tract samples included nasopharyngeal aspirates, nasopharyngeal swabs, throat swabs, and combined nasopharyngeal/throat swabs. Lower tract samples included lung aspirates, bronchoalveolar lavages, endotracheal aspirates, bronchial brushing, and sputum samples. ‘Other’ samples included respiratory samples without definite categorisation. Abbreviations: IQR: inter quartile range, IRSD: index of relative socio-economic disadvantage, N/A: Not Applicable. (DOCX) [file pone.0313504.s006.docx]

|  |  | **Any Virus Positive** | **Single Virus Positive** | **Dual Virus Positive** | **Triple Virus Positive** | **Four Virus Positive** | **Five Virus Positive** | **Six Virus Positive** | ***Total Multi Virus Positive*** |
| --- | --- | --- | --- | --- | --- | --- | --- | --- | --- |
|  | **Count**  **(% Positive)** | 20510 (100) | 15082 (73.5) | 4284 (20.9) | 904 (4.4) | 194 (0.9) | 39 (0.2) | 7 (0.0) | *5428 (26.5)* |
| **Sex** | **Male (%)** | 11828 (57.7) | 8585 (56.9) | 2554 (59.6) | 547 (60.5) | 113 (58.2) | 24  (61.5) | 5  (71.4) | 3243  (59.7) |
| **Age (months)** | **Median (IQR)** | 14 (5-38) | 15 (4-45) | 12 (6-25) | 13 (8-20) | 14 (10-19) | 16 (13-22) | 19 (16-35) | *13 (6-23)* |
| **Age Group** | **<6 months** | 5490 (26.8) | 4287 (28.4) | 1051 (24.5) | 132 (14.6) | 20 (10.3) | - | - | *1203 (22.2)* |
|  | **6-11 months** | 3583 (17.5) | 2296 (15.2) | 953 (22.2) | 272 (30.1) | 55 (28.4) | 7 (17.9) | - | *1287 (23.7)* |
|  | **1-4 years** | 8183 (39.9) | 5623 (37.3) | 1939 (45.3) | 467 (51.7) | 116 (59.8) | 31 (79.5) | 7 (100.0) | *2560 (47.2)* |
|  | **5-9 years** | 2109 (10.3) | 1845 (12.2) | 242 (5.6) | 20 (2.2) | 1 (0.5) | 1 (2.6) | - | *264 (4.9)* |
|  | **10-15 years** | 1145 (5.6) | 1031 (6.8) | 99 (2.3) | 13 (1.4) | 2 (1.0) | - | - | *114 (2.1)* |
| ***IRSD** | **Lowest Quintile** | 3906 (19.0) | 2822 (18.7) | 865 (20.2) | 173 (19.1) | 37 (19.1) | 8 (20.5) | 1 (14.3) | *1084 (20.0)* |
|  | **2nd Quintile** | 3204 (15.6) | 2368 (15.7) | 681 (15.9) | 121 (13.4) | 24 (12.4) | 9 (23.1) | 1 (14.3) | *836 (15.4)* |
|  | **3rd Quintile** | 2300 (11.2) | 1702 (11.3) | 470 (11.0) | 101 (11.2) | 22 (11.3) | 5 (12.8) | - | *598 (11.0)* |
|  | **4th Quintile** | 4518 (22.0) | 3280 (21.7) | 966 (22.5) | 210 (23.2) | 51 (26.3) | 8 (20.5) | 3 (42.9) | *1238 (22.8)* |
|  | **Highest Quintile** | 6188 (30.2) | 4616 (30.6) | 1221 (28.5) | 283 (31.3) | 57 (29.4) | 9 (23.1) | 2 (28.6) | *1572 (29.0)* |
|  | **Overseas (N/A)** | 394 (1.9) | 294 (1.9) | 81 (1.9) | 16 (1.8) | 3 (1.5) | - | - | *100 (1.8)* |
| **Discharge Location** | **Inpatient** | 14262 (69.5) | 10717 (71.1) | 2806 (65.5) | 590 (65.3) | 126 (64.9) | 18 (46.2) | 5 (71.4) | *3545 (65.3)* |
|  | **Emergency Department** | 5243 (25.6) | 3573 (23.7) | 1299 (30.3) | 287 (31.7) | 63 (32.5) | 19 (48.7) | 2 (28.6) | *1670 (30.8)* |
|  | **Outpatient or Other Location** | 1005 (4.9) | 792 (5.3) | 179 (4.2) | 27 (3.0) | 5 (2.6) | 2 (5.1) | - | *213 (3.9)* |
| **^Length of Stay (days)** | **Median (IQR)** | 2 (1-5) | 2 (1-6) | 2 (1-4) | 2 (1-3) | 1 (1-3) | 1 (0-2) | 1 (1-3) | *2 (1-4)* |
| **Time to Sampling (days)** | **Median (IQR)** | 1 (0-1) | 1 (0-2) | 0 (0-1) | 0 (0-1) | 0 (0-1) | 0 (0-0) | 1 (0-1) | *0 (0-1)* |
| **^$^Respiratory Tract Sampling Location** | **Upper Tract** | 19929 (97.2) | 14601 (96.8) | 4202 (98.1) | 888 (98.2) | 193 (99.5) | 38 (97.4) | 7 (100.0) | *5328 (98.2)* |
|  | **Lower Tract** | 509 (2.5) | 418 (2.8) | 75 (1.8) | 14 (1.5) | 1 (0.5) | 1 (2.6) | - | *91 (1.7)* |
|  | **Other** | 72 (0.4) | 63 (0.4) | 7 (0.2) | 2 (0.2) | - | - | - | *9 (0.2)* |
